# Defining Mediterranean and Black Sea Biogeochemical Subprovinces and Synthetic Ocean Indicators Using Mesoscale Oceanographic Features

**DOI:** 10.1371/journal.pone.0111251

**Published:** 2014-10-31

**Authors:** Anne-Elise Nieblas, Kyla Drushka, Gabriel Reygondeau, Vincent Rossi, Hervé Demarcq, Laurent Dubroca, Sylvain Bonhommeau

**Affiliations:** 1 Unité Mixte Recherche Ecosystèmes Marins Exploités 212, Institut Français de Recherche pour l'Exploitation de la Mer (IFREMER), Sète, France; 2 Applied Physics Laboratory, University of Washington, Seattle, Washington, United States of America; 3 Center for Macroecology, Evolution and Climate, National Institute for Aquatic Resources, Technical University of Denmark (DTU Aqua), Charlottenlund, Copenhagen, Denmark; 4 Instituto de FÍsica Interdisciplinary Sistemas Complejos, Institute for Cross-Disciplinary Physics and Complex Systems, (CSIC-UIB), Campus Universitat de les Illes Balears, Palma de Mallorca, Spain; 5 Unité Mixte de Recherche Ecosystèmes Marins Exploités 212, Institut de Recherche pour le Développement (IRD), Sète, France; 6 European Commission, Joint Research Center, Institute for Environment & Sustainability, Water Resources, Ispra, Italy; Università della Calabria, Italy

## Abstract

The Mediterranean and Black Seas are semi-enclosed basins characterized by high environmental variability and growing anthropogenic pressure. This has led to an increasing need for a bioregionalization of the oceanic environment at local and regional scales that can be used for managerial applications as a geographical reference. We aim to identify biogeochemical subprovinces within this domain, and develop synthetic indices of the key oceanographic dynamics of each subprovince to quantify baselines from which to assess variability and change. To do this, we compile a data set of 101 months (2002–2010) of a variety of both “classical” (i.e., sea surface temperature, surface chlorophyll-*a*, and bathymetry) and “mesoscale” (i.e., eddy kinetic energy, finite-size Lyapunov exponents, and surface frontal gradients) ocean features that we use to characterize the surface ocean variability. We employ a k-means clustering algorithm to objectively define biogeochemical subprovinces based on classical features, and, for the first time, on mesoscale features, and on a combination of both classical and mesoscale features. Principal components analysis is then performed on the oceanographic variables to define integrative indices to monitor the environmental changes within each resultant subprovince at monthly resolutions. Using both the classical and mesoscale features, we find five biogeochemical subprovinces for the Mediterranean and Black Seas. Interestingly, the use of mesoscale variables contributes highly in the delineation of the open ocean. The first axis of the principal component analysis is explained primarily by classical ocean features and the second axis is explained by mesoscale features. Biogeochemical subprovinces identified by the present study can be useful within the European management framework as an objective geographical framework of the Mediterranean and Black Seas, and the synthetic ocean indicators developed here can be used to monitor variability and long-term change.

## Introduction

Growing pressure on the European marine environment has led to an increasing demand for comprehensive evaluation and monitoring programs [1]–[4]. The Mediterranean and Black Seas are ecologically- and economically-important semi-enclosed seas characterized by highly specific biogeochemcial, oceanographic, and environmental conditions that have resulted in pronounced endemism of exploited marine species [5]–[7]. The Mediterranean Sea is commonly divided into two basins, east and west, which each have specific hydrological conditions and marked seasonal cycles [8]. Recently, the International Panel on Climate Change has designated the Mediterranean as one of the most perturbed marine ecosystems of the global ocean, as both deep and surface environments show significant change in the open seas, coastal, benthic and neritic areas [9]–[11]. In addition, it is undergoing increasing anthropogenic pressure, including pollution, overfishing, and habitat loss via coastal development [1], [7], [12].

In this context, the European Union has recently adopted the Integrated Maritime Policy framework for the protection of European Seas; the primary objective of which is to achieve environmentally healthy waters by 2020 [1]–[4]. The first step toward the goal of healthy waters and the aim of this study is to identify an objective spatial partitioning in the Mediterranean and Black Seas, where environmental conditions are homogeneous, to act as a framework for marine zoning [13], [14], for ecological management [15], [16], as well as to determine baseline conditions which can then be used to effectively monitor variability and change.

Marine bioregionalization aims to identify unique and homogeneous biogeochemical partitions delineated by observable frontiers, such as frontal structures. This discipline, recently redefined by [17], is based on objective statistical methodologies and has been applied in several regions of the global ocean at several different scales [18]–[21]. However, owing to the complex hydrodynamics [22], [23] and the important influence of mesoscale activity on biogeochemcial processes [24]–[26], bioregionalization of the Mediterranean and Black Seas remains difficult.

Marine bioregionalizations are classically performed on oceanographic features that are thought to be representative of the oceanographic and biogeochemical structure of a region; for example, sea surface temperature (SST), bathymetry and surface chlorophyll-*a* (chl) [20], [27], [28]. However, mesoscale processes must also be important for defining biogeochemical partitions as they are known to impact ocean productivity, including the spatial distribution and stocks of chlorophyll-*a* [29], [30]; and basin-scale circulation and its mesoscale variability have been shown to be crucial in delineating hydrodynamical regions [31].

Previous studies have used single or multivariate analyses to derive regions of similar features in the Mediterranean Sea, including classical oceanographic indicators (i.e., chl [8]; SST, chl, sea surface salinity, and bathymetry [32]), bio-physical indicators, such as Ekman pumping, nutrient concentration, euphotic depth, and stratification [32]; and exploited fish distributions and biodiversity [7]. Recently, the Mediterranean Sea was subdivided into several hydrodynamical provinces delineated by multi-scale oceanic frontal structures in order to assess the ecological connectivity of the whole basin [31].

In this study, we derive objective biogeochemical subprovinces (sensu [33]) of the Mediterranean and Black Seas based on multivariate analyses of classical oceanographic features (SST, chl, and bathymetry), mesoscale features (eddy kinetic energy (EKE), SST and chl surface fronts, finite-size Lyapunov exponents (FSLE), and the Okubo-Weiss (OW) parameter), and a combination of both classical and mesoscale features. We also quantify the stability of the boundaries between the biogeochemical subprovinces in time and space. Synthetic oceanographic indices for the subprovinces are then extracted to act as baseline indicators, using principal components analysis (PCA), similar to the multivariate ocean-climate indices recently developed by [34]. Finally, we examine the temporal variability of these indices and their relationships with large-scale climate indices. The biogeochemical subprovinces identified in this study and the time series of their synthetic indicators could become important tools within the European management framework for assessing the environmental variability and change within the Mediterranean Sea.

## Materials and Methods

### Data

We used daily 4-km, version 5 Advanced Very High Resolution Radiometer pathfinder SST (1982–2012) available at http://www.nodc.noaa.gov/sog/pathfinder4km/. Chl data were taken from the National Aeronautics and Space Administration's daily 4-km level-3 Moderate Resolution Imaging Spectroradiometer daily data set (2002–2010) available at http://oceancolor.gsfc.nasa.gov/. We extracted weekly 1/3° (i.e., about 33 km at these latitudes) Ssalto/Duacs sea level anomalies and geostrophic velocity anomalies (u,v) computed and distributed by Aviso (1992–2012), with support from the Centre National d'Études Spatiales (http://www.aviso.oceanobs.com/duacs/). The bathymetry of the Mediterranean basin was extracted from the ETOPO1 database hosted on the National Oceanic and Atmospheric Administration's website at ∼4 km resolution using the *getNOAA.bathy* function (marmap package, http://cran.r-project.org/). For consistency between variables, analyses were performed for data between May 2002 and November 2010, totaling 101 months.

### Oceanographic indices

Several features were derived by further processing the remotely-sensed data. SST and chl fronts were computed with the gradient method, using a common sobel operator (e.g., [35], [36]). These continuous values indicate the frontal intensity between water masses.

Using geostrophic velocity anomalies, we calculated several indicators of mesoscale ocean features. These data were used to derive backward-calculated FSLEs, which measure the horizontal mixing and dispersion in the ocean [37] and help to detect mesoscale Lagrangian coherent structures of ecological significance (e.g., [38]). FSLEs are defined as λ(**x**,t,δ_0_,δ*_f_*)  =  (*1/τ*)log(δ_0_/δ*_f_*) where λ(**x**,t,δ_0_,δ*_f_*) is the FSLE at position **x** and time t with an initial separation distance from **x** of δ_0_ and final separation distance from **x** of δ*_f_*. Here, we assign δ_0_ to be 0.04 degrees and δ*_f_* to be 0.6 degrees, and a time interval, τ, of 200 days, following the FSLE parameterizations of the Center for Topographic studies of the Ocean and Hydrosphere (http://ctoh.legos.obs-mip.fr/products/submesoscale-filaments/fsle-description), allowing us to detect mesoscale structures of <100 km, an appropriate scale for these seas [37]. Geostrophic velocity anomalies were also used to compute EKE, *(u^2^+v^2^)/2*, which is an indicator of the intensity of the eddy activity. Finally, we use geostrophic velocity anomalies to compute the OW parameter [39], [40], W = s^2^_n_+s^2^_s_+ω^2^, where s_n_ and s_s_ are the normal and shear components of strain, and ω is the relative vorticity. This parameter is used to identify regions of high vorticity (W<0), which are likely related to the cores of mesoscale ocean eddies [41].

Monthly mean time series (2002–2010) were derived for all variables (except bathymetry) for the region including the Mediterranean and the Black Seas (30°N to 47°N, −6°E to 42°E) and regridded at 4 km resolution. We natural log transformed chl, chl fronts and bathymetry in order to stabilize their variance as their values can span several orders of magnitude.

### Spatio-temporal multivariate k-means cluster analysis

**Figure 1:**
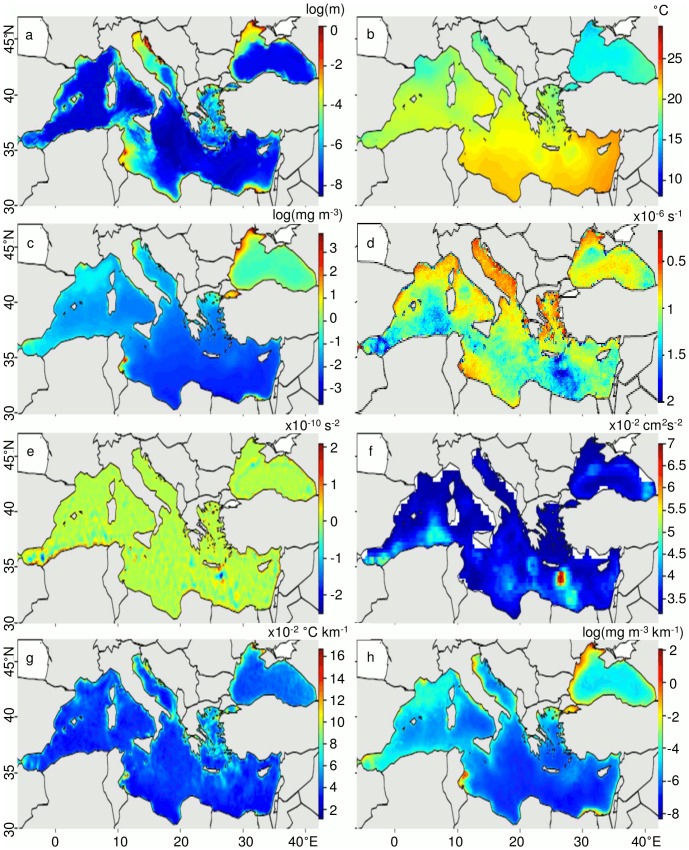
Time-averages of all oceanographic variables collected for the Mediterranean Sea. Variables include a) natural log-transformed bathymetry, b) sea surface temperature (SST), c) natural log-transformed chlorophyll-*a* (chl), d) finite-size Lyapunov exponents (FSLE), e) Okubo-Weiss parameter (OW), f) eddy kinetic energy (EKE), g) SST surface frontal gradients, and h) natural log-transformed chl surface frontal gradients.

**Figure 2:**
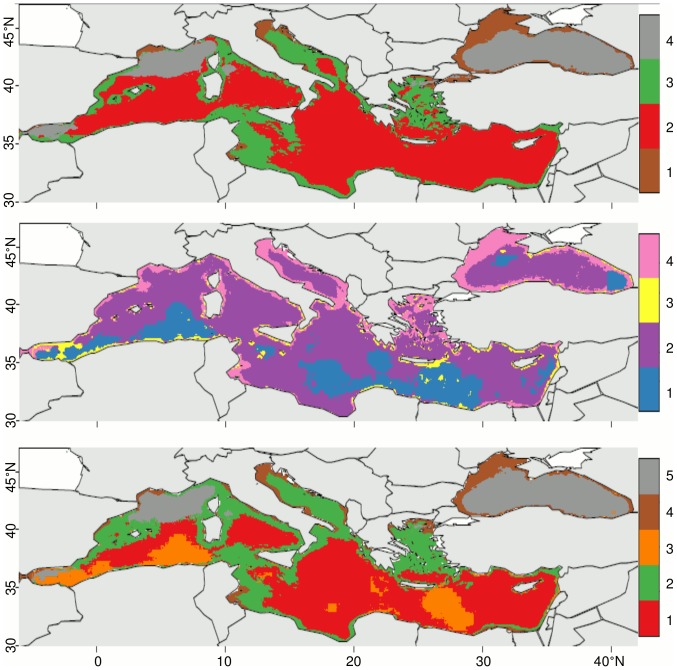
Biogeochemical subprovinces of the Mediterranean and Black Seas. Subprovinces for the (a) “classical”, (b) “mesoscale”, and (c) “full” multivariate arrays using a 5% threshold for the explained sum of squares to define the optimal number of subprovinces (see text).

Multivariate arrays were created from time-averages of the monthly scaled oceanographic indices (Figure 1) and combined into “classical” (i.e., SST, chl, and bathymetry), “mesoscale” (i.e., FSLE, OW, EKE, and SST and chl surface fronts), and “full” arrays (i.e., all features). After initial tests, k-means (*kmeans*, stats package, http://cran.r-project.org/; [42]) was determined to be the most robust cluster analysis algorithm to objectively classify biogeochemical subprovinces. This partitioning method, using Euclidean distances, assigns data points to *k* clusters and minimizes the sum of squares between the data points to cluster centre. With this algorithm, *k* must be defined *a priori*. In order to define *k*, we bootstrap (1000 times) *k* between 2 and 30. The between-clusters sum of squares is then divided by the total sum of squares to find the explained sum of squares. Arbitrary 1% and 5% thresholds are defined (Figure S1 in File S1), which we used to define the optimal *k* for the three multivariate arrays (Table 1), whereby the explained sum of squares for each additional *k* increases by less than 1% and 5%, respectively. K-means analyses were then performed on each array using the optimal *k* for both threshold levels (1%; Figure S2 in File S1 and 5%; Figure 2). The resultant clusters were defined as the biogeochemical subprovinces of the Mediterranean and Black Seas as a subdivision of the Mediterranean provinces defined by [33].

**Table 1:** Optimal number of clusters, *k*, for each multivariate array for the 1% and 5% threshold levels obtained by bootstrapping 1000 times the k-means analysis on *k* between 2 and 30.

Array	Threshold	
	1%	5%
Classical	9	4
Mesoscale	14	4
Full	13	5

**Figure 3:**
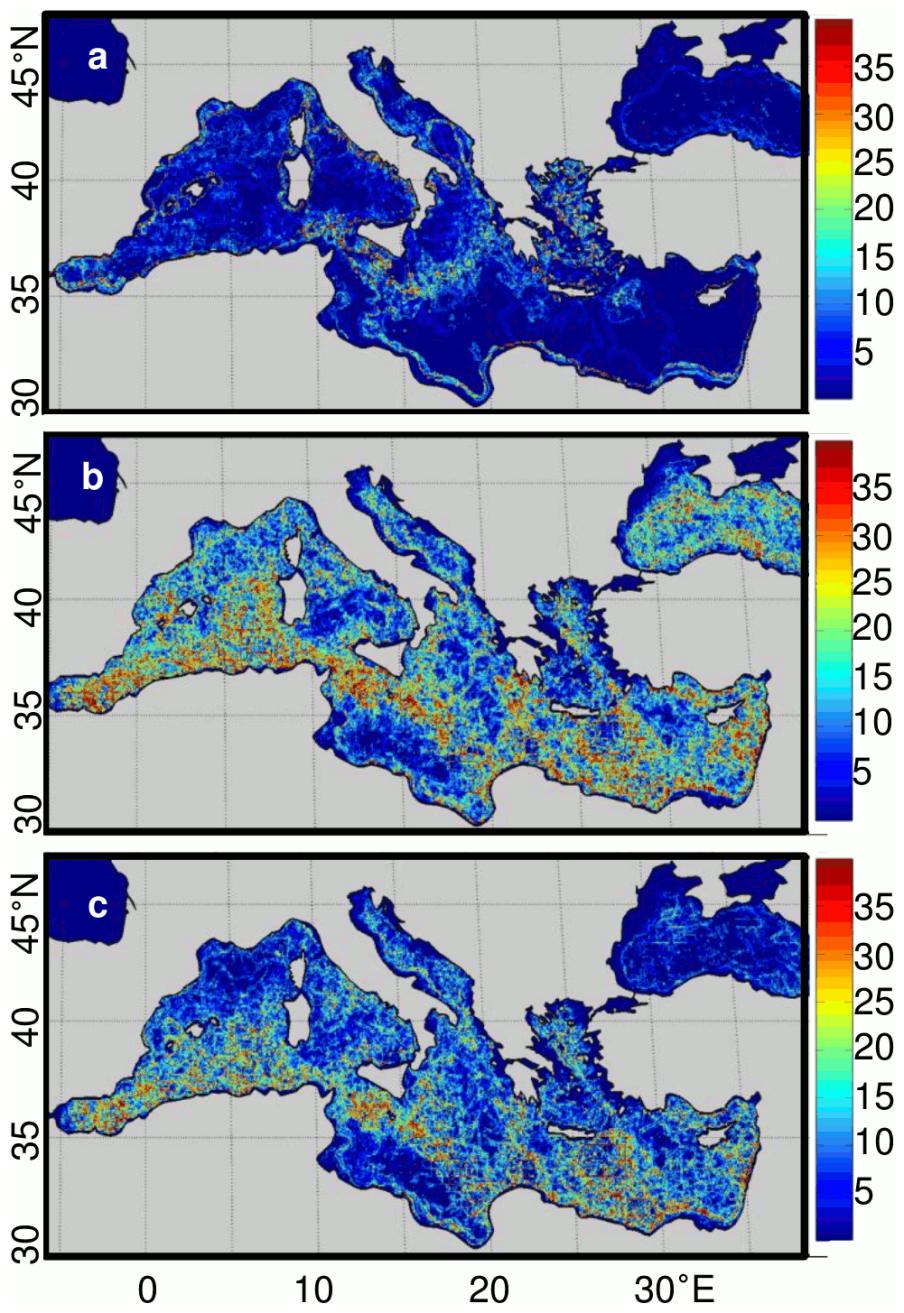
Spatial stability of the borders of biogeochemical subprovinces. Stability plots for the (a) classical, (b) mesoscale, and (c) full multivariate arrays. K-means analyses, using the k found in the time-averaged analyses (Table 1), are performed on the multivariate arrays at monthly time steps for the 101 months of the data set and using a 5% threshold of the explained sum of squares to define the optimal number of subprovinces (see text). Spatial stability is represented as the percentage of time that a boundary of the biogeochemical subprovinces is found at a particular pixel over the 101 months of the data set. Red colors indicate stable borders.

To investigate the spatial stability of the subprovinces through time, we used the optimal *k* values found for each of the three multivariate arrays for both the 1% and 5% threshold levels, and performed a k-means analysis on each of the multivariate arrays for every month of the data set (n = 101 months). Then, based on an adaptation of the effectiveness test implemented by [43], the temporal stability of each geographical cell is computed as the percentage of time that a geographical cell is considered as a boundary between two clusters at each temporal step (Figure 3, Figure S3 in File S1).

### Development of synthetic indices through PCA

In order to develop synthetic indices of the oceanographic indicators for each biogeochemical subprovince, we extracted the scaled and centered monthly time series of each oceanographic variable (except bathymetry) for each pixel within each biogeochemical subprovince. Although bathymetry is important for determining the biogeochemical subprovinces, it does not vary in time and was not included in the PCA. The strong seasonal cycle observed in all time-series was removed before performing the PCA as this signal swamps both the lower- and higher-frequencies of the time series (e.g., [44]). We then performed a PCA for each biogeochemical subprovince with an individual being the monthly value of each oceanographic variable for each pixel. We used the common cutoff of eigenvalues >1 to retain the unrotated principal components (PCs) (Table S1). We then took the monthly mean of the retained PCs over all the pixels, and used these as the synthetic indices of each biogeochemical subprovince.

Finally, we investigated the mode of temporal variability of these synthetic indices. Spectra were calculated to show the variability of each time-series. Lagged correlations were then investigated between time-series and monthly anomalies of four independent large-scale climate indices known to influence Mediterranean Sea dynamics [45], [46]: North Atlantic Oscillation (NAO), the East Atlantic pattern (EA), the East Atlantic-West Russia pattern (EAWR), and the Scandinavian pattern (SCAND). These indices were computed by the National Oceanic and Atmospheric Administration/Climate Prediction Center.

## Results

**Figure 4:**
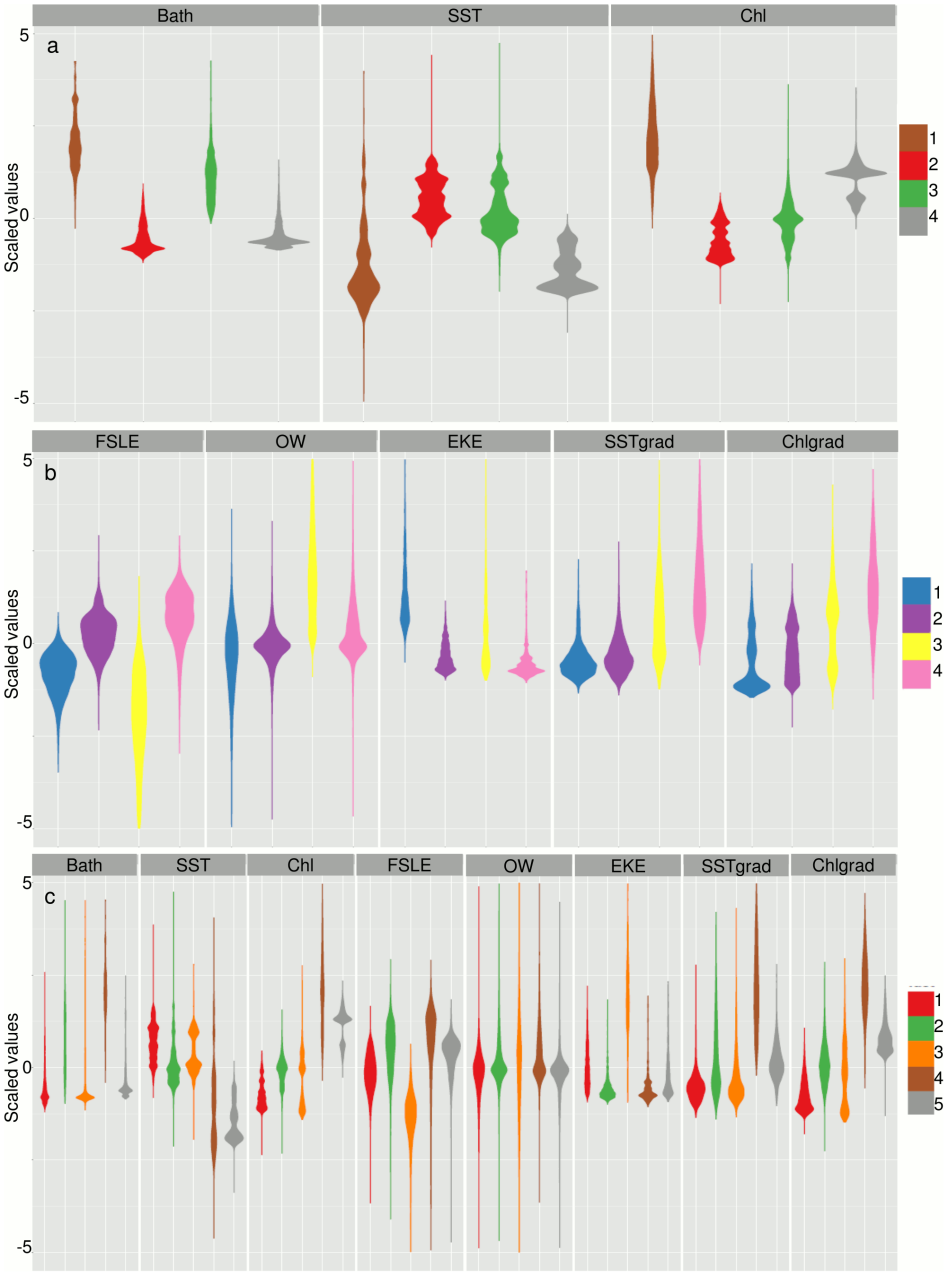
Violin plots of the scaled values for each oceanographic variable. Violin plots for the (a) classical, (b) mesoscale, and (c) full multivariate arrays. Colors indicate biogeochemical subprovinces. The mean of each variable is represented by the bulge in the violin and its variability is indicated by the tails. Here, positive bathymetry is shallow and negative bathymetry is deep. Variable abbreviations are as follows: sea surface temperature (SST), chlorophyll-*a* concentration (chl), finite-sized Lyapunov exponents (FSLE), the Okubo-Weiss parameter (OW), eddy kinetic energy (EKE), and SST and chl frontal gradients (SSTgrad, and chlgrad, respectively). Colorbars represent subprovinces.

**Figure 5:**
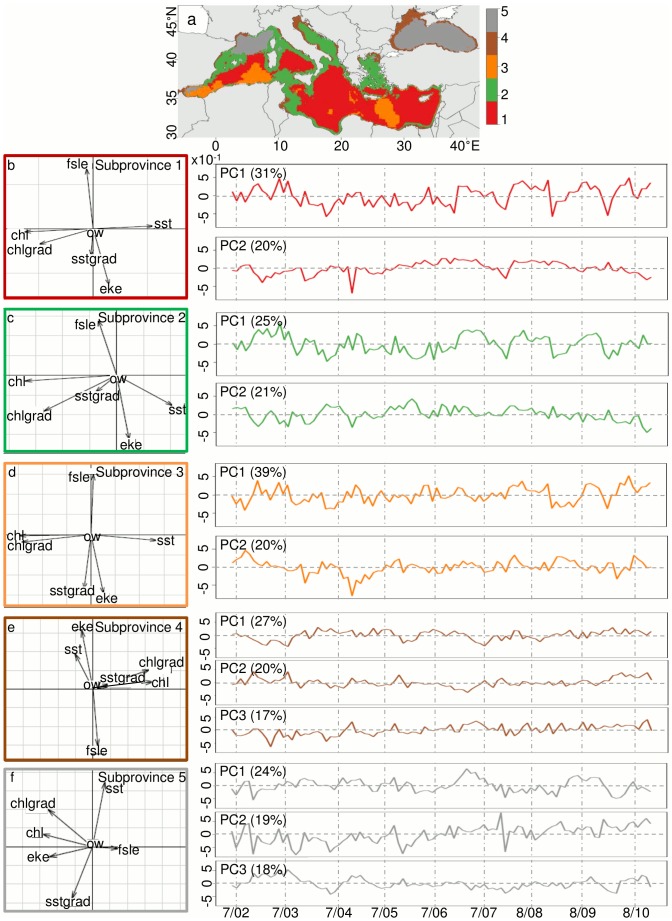
Principal component (PC) analysis for the biogeochemical subprovinces of the Mediterranean Sea. The (a) biogeochemical subprovinces of the Mediterranean Sea, as defined by the 5% threshold for the full multivariate array, and the (b–f) PC analysis arrow plots and time series of the retained PCs for each of the subprovinces, derived from the mean of all pixels in the subprovince for each month of the data set. Arrows that align well with an axis are well-explained by that axis. The longer the arrow, the more it contributes to explaining the variability of an axis. Variable abbreviations are as in Figure 4.

**Figure 6:**
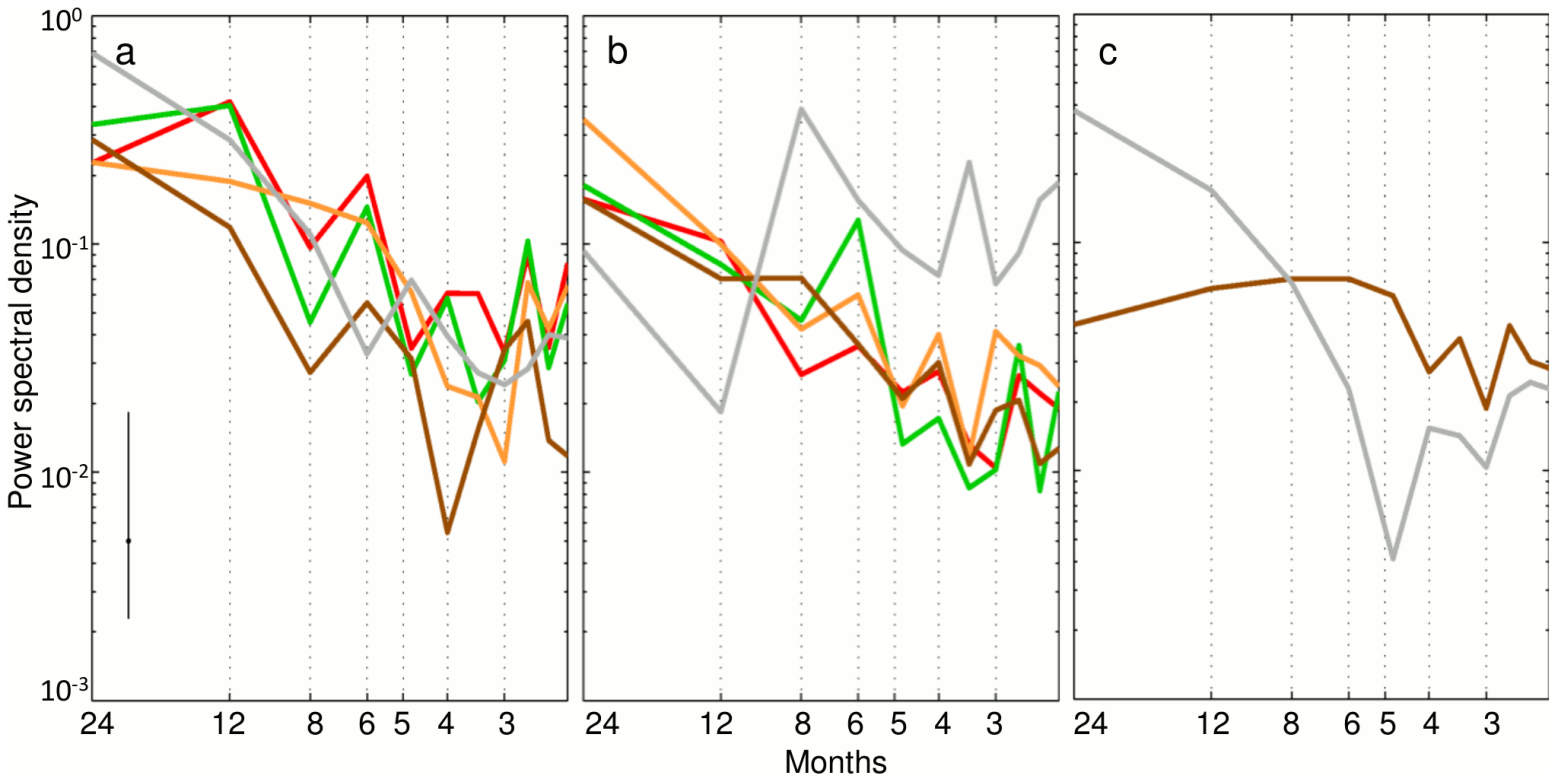
Spectral energy plots. Spectral energy plots for (a) principal component (PC) 1, (b) PC2, and (c) PC3 as retained for the different biogeochemical subprovinces computed for 101 months, having removed the seasonal signal. Peaks are indicated for all PCs at interannual frequencies. The error bar in the bottom-left indicates the 95% significance level.

At the 5% threshold level, we find four subprovinces for the classical oceanographic features, four subprovinces for the mesoscale features and five subprovinces for the full combination of features (Table 1, Figure 2). Overall, the “classical” array (i.e., defined using the set of classical features) has the most stable boundaries in time and space (Figure 3a), while the boundaries for the “mesoscale” array (i.e., defined using the set of mesoscale features only) are highly variable (Figure 3b), as are the boundaries of the “full” array (i.e., defined using both classic and mesoscale features) (Figure 3c). This indicates that the apparent stability found for the classical array is not representative of the “true” *in-situ* variability of the oceanic environment. The 5% threshold identifies fewer biogeochemical subprovinces than the 1% threshold (Table 1; Figure 2; Figure S2 in File S1), which, in addition to higher stability (Figure 3; Figure S3 in File S1), makes them easier to monitor in a management context. The full array at the 5% threshold was finally determined to be the most useful for management purposes, as the subprovinces are realistic and inclusive of both classical and mesoscale features (Figure 2, 3, 4). We perform a PCA for the subprovinces defined for the 5% threshold of the full array to derive synthetic time series (Table S1, Figure 5). The first two PCs are retained for full subprovinces 1–3 (explaining up to 39% and 21% of the variance for PC1 and PC2, respectively), and the first three PCs are retained for full subprovinces 4–5 (explaining up to 27%, 20%, and 18% of the variance for PC1, PC2 and PC3, respectively). We find strong low frequency (interannual) energy for the first two PCs in all full subprovinces (Figure 6), with the PCs of subprovince 5 being particularly different from the rest, especially PC2. We do not find any meaningful correlations to large-scale climate indices for any of the retained PCs (Table S2).

The stability of the boundaries for a given biogeochemical subprovince indicates whether the subprovince is representative of the local hydrological and biogeographical conditions, with higher stability giving greater credence. We found that the stability of the borders of the biogeochemical subprovinces defined by the 5% threshold (Figure 3) were more stable in terms of time and space than those of the 1% threshold (Figure S3 in File S1). The boundaries of the biogeochemical subprovinces of the classical array are very clearly defined and spatially stable through time (Figure 3a), with the greatest stability of boundaries found at the coastal isobaths (Figure 1a). Greater variability in the boundaries occurs through the Strait of Sicily and in the Aegean Seas, which correspond to high spatial bathymetric variability. The variability in biogeochemical subprovince boundaries observed in the Ionian Sea appears to correspond to a latitudinal transition of SST (Figure 1b). The boundaries for the mesoscale array are extremely variable (Figure 3b), with boundaries shifting significantly for each month of the data set. Especially high variability occurs in the southern parts of both the eastern and western basins, including the Alboran Sea, Algerian Basin, through the Strait of Sicily and south of Malta, where mesoscale activity is particularly high (Figure 1d,e,f).

Despite this spatial variability, we find that the subprovinces derived from the classical and mesoscale features are often grouped in the same manner. For example, the coastal Gulf of Lions, the northern Adriatic Sea, the Aegean Sea, the coastal Gulf of Gabes, the coast near the Suez Canal, and the western Black Sea are consistently grouped together in all three multivariate arrays (classical subprovince 1, mesoscale subprovince 4, full subprovince 4; Figure 2). These subprovinces are characterized by shallow bathymetry, generally low but variable SSTs, and high and variable chl (Figure 4a, c; Table 2). In terms of mesoscale features, these subprovinces have relatively consistently high FSLE, indicating low horizontal mixing, low EKE, and high SST and chl frontal gradients (Figure 4b, c; Table 2). Offshore of the Gulf of Lions and the offshore Black Sea are also commonly grouped by the three multivariate arrays (classical subprovince 4, mesoscale subprovince 2, full subprovince 5), and are characterized by deep bathymetry, low and relatively constant SSTs, and high chl (Figure 4a, c; Table 2). This subprovince has high FSLE, again indicating low horizontal mixing, low EKE (Figure 4b, c; Table 2), and high chl frontal gradients for the full subprovince 5 (Figure 4c; Table 2). The Strait of Gibraltar/Alboran Sea is grouped mainly by classical subprovinces 3 and 4 (Figure 2a, c), but is more variably characterized by mesoscale features (Figure 1, Figure 4). The majority of the southern parts of the eastern and western basins are grouped together (classical subprovince 2, mesoscale subprovince 2, full subprovince 1), and are characterized by deeper bathymetry, higher SSTs, low chl, and low mesoscale activity (Figure 4; Table 2). The eastern and western basin are divided by the Strait of Sicily for the classical and full subprovinces (Figure 2a, c) due to relatively shallow bathymetry (Figure 4a,c; Table 2). Mesoscale subprovince 1 appears related to full subprovince 3, which both occupy the southern basin, and are characterized by low FSLE (strong mixing), low and variable OW (indicative of eddy cores), high EKE, and low SST and chl frontal gradients (Figure 4b; Table 2). This high EKE is especially apparent in full subprovince 3, south of Crete in the Levantine Sea (e.g., the wind-driven Ierapetra anticyclonic gyre), in the Algerian Basin, and in the Alboran Sea (Figure 1f, 2c).

**Table 2:** Mean and standard deviations of the environmental variables for each biogeochemical subprovince defined by the 5% threshold of the classical, mesoscale, and full multivariate arrays over the 101 months of the data set, including sea surface temperature (SST, °C), chlorophyll-*a* concentration (chl; mg m^−3^), finite-sized Lyapunov exponents (FSLE; s^−1^), the Okubo-Weiss parameter (OW; s^−2^), eddy kinetic energy (EKE; cm^2^ s^−2^), and SST and chl frontal gradients (SSTgrad; °C km^−1^, and chlgrad; mg m^−3^ km^−1^, respectively).

Subprovince	SST	Chl	Bath	EKE	FSLE	OW	SSTgrad	Chlgrad
Classical								
1	17.04±6.66	0.19±0.24	−56.75±73.12					
2	20.85±4.41	0.15±0.12	−2133.17±945.04					
3	20.19±4.55	0.24±0.26	−203.08±178.70					
4	16.98±6.01	0.65±0.59	−1786.03±658.50					
Mesoscale								
1				0.042±0.012	−1.32e-06±7.01e-07	−7.36e-12±8.69e-11	0.038±0.024	0.005±0.015
2				0.035±0.004	−1.00e-06±4.91e-07	1.44±3.59e-11	0.039±0.025	0.006±0.017
3				0.037±0.008	−1.84e-06±1.47e-06	4.99e-11±8.33e-11	0.052±0.035	0.026±0.094
4				0.034±0.003	−8.69e-07±5.67e-07	9.94e-12±3.34e-11	0.065±0.043	0.086±0.210
Full								
1	21.13±4.38	0.13±0.09	−2152.13±1021.32	0.036±0.005	−1.12e-06±5.5e-07	−7.62e-13±4.87e-11	0.03±0.02	0.003±0.004
2	19.95±4.57	0.25±0.005	−609.96±648.89	0.034±0.003	−9.39e-07±5.6e-07	8.09e-12±3.74 e-11	0.046±0.029	0.008±0.018
3	20.54±4.10	0.20±0.25	−2210.62±962.95	0.044±0.014	−1.57e-06±1.05e-06	4.00e-12±1.078e-10	0.040±0.026	0.008±0.026
4	17.80±6.48	1.91±2.84	−77.36±132.71	0.034±0.003	−8.95e-07±6.95e-07	1.18e-11±3.53e-11	0.072±0.047	0.155±0.279
5	16.87±6.09	0.65±0.49	−1774.19±722.23	0.035±0.005	−9.69e-07±4.66e-07	−3.76e-13±3.82e-11	0.045±0.030	0.017±0.045

In general, we find that classical variables for the full subprovinces primarily explain the first PC, and mesoscale variables for the full subprovinces generally explain the second PC (Figure 5b–f arrow plots, left panels; Table 3), with full subprovince 5 exhibiting much different patterns for all PCs than the other full subprovinces. For each PC, the variables having a correlation value >0.5 with a particular PC time series are deemed significant (a necessarily subjective cutoff value relevant only to this data set; Table 3). We find that the first two axes are typically divided between classical (chl and SST, as well as chl frontal gradients; PC1), and mesoscale features (typically FSLE and EKE, and SST frontal gradients for subprovince 3; PC2) (Figure 5b–d arrow plots), while OW does not explain any axis (Table 3). Chl and chl frontal gradients are highly correlated (r = 0.92), explaining the alignment of this mesoscale feature with classical features on the first axis. FSLE and EKE are also highly negatively correlated for each subprovince (Figure 5b–d arrow plots). PC1 for subprovinces 1, 2, and 3 are significantly correlated (p<0.001), with relatively high correlation coefficients (p<0.05, r = 0.61 to 0.8; Table S2), indicating that their classical features vary in the same manner. PC2 for subprovinces 1 and 2 are also significantly correlated (p<0.05, r = 0.58; Table S2).

**Table 3:** Correlation matrix of the first four principal components (PC) analyzed for the full multivariate array, as defined by the 5% threshold, including sea surface temperature (SST), chlorophyll-*a* concentration (chl), eddy kinetic energy (EKE), finite-sized Lyapunov exponents (FSLE), the Okubo-Weiss parameter (OW), and SST and chl frontal gradients (SSTgrad and chlgrad, respectively).

Subprovince	PC	Variance explained (%)	Eigenvalues of environmental variables						
			SST	Chl	EKE	FSLE	OW	SSTgrad	Chlgrad
1	1	31	**0.56**	**−0.64**	0.15	−0.06	0	−0.02	**−0.5**
	2	20	0.03	−0.04	−**0.64**	**0.68**	0	−0.31	−0.18
	3	16	−0.1	0.03	0.24	−0.2	0	−0.94	−0.02
2	1	25	0.42	**−0.69**	0.09	−0.14	0	−0.16	**−0.54**
	2	21	−0.31	−0.06	**−0.65**	**0.56**	0	−0.18	−0.37
	3	16	0.1	0.19	0.12	0.01	0	−0.96	0.14
3	1	39	**0.55**	**−0.6**	0.1	0.02	0	−0.06	**−0.58**
	2	20	−0.06	−0.01	**−0.58**	**0.6**	0	**−0.54**	−0.07
	3	15	0	0.04	0.12	−0.59	0	−0.79	0.04
4	1	27	−0.21	**0.69**	−0.13	0.06	0	0.19	**0.65**
	2	20	0.39	0.08	**0.64**	**−0.62**	0	0.05	0.21
	3	17	**0.8**	−0.15	−0.18	0.4	0	0.24	0.28
5	1	24	0.14	**−0.57**	**−0.5**	0.3	0	−0.24	**−0.51**
	2	19	**0.7**	0.14	−0.11	−0.02	0	**−0.56**	0.4
	3	18	0.23	−0.38	0.41	**−0.73**	0	−0.08	−0.3

Variables with correlation values >0.5 (bold) are deemed to significantly contribute to each PC.

Three axes are retained for subprovinces 4 and 5, as the eigenvalues for their PC3 were >1 (Figure 5e, f). The classical and mesoscale features are not as clearly divided by the retained axes for subprovinces 4 and 5 as they are for the first three subprovinces. The first axis for subprovince 4 is primarily explained by chl and chl frontal gradients and the second axis is explained by FSLE and EKE, similar to subprovinces 1–3, but the third axis is primarily explained by SST (Table 3). Subprovince 5 is even more different as the first and second axes are explained by a mix of classical and mesoscale features: PC1 is primarily explained by chl, chl frontal gradients and EKE, PC2 is explained by SST and SST frontal gradients, and PC3 is explained by FSLE.

The PC time series represent synthetic indices of oceanographic dynamics for each full subprovince (Figure 5b–f, right panels). After removing the dominant seasonal cycle, power spectra reveal that low-frequency (interannual) variability dominates PC1 for all subprovinces (Figure 6a) as well as PC2 for all subprovinces except subprovince 5 (Figure 6b). Subprovince 5 has distinctive spectral characteristics, with no high-frequency peak for PC1 (Figure 6a) and no decrease in energy at high frequencies for PC2. Instead, PC2 has a strong peak at a period of 8 months (Figure 6b). Subprovince 5 also has a strong low-frequency signal for PC3, but no significant signals are found for PC3 of subprovince 4 (Figure 6c).

We find six out of 48 significant correlations between the retained PCs and large-scale climate indices (r = −0.2 to 0.27 with p-value <0.05). However, this could be due to multiple testing of time series. Since the correlations were low, we did not correct the p-values for multiple testing and we consider that there is no major links between the PCA axes and climatic indices. Lagged correlations between the PCs and the climate indices were also considered but did not render stronger relationships (Table S2).

## Discussion

Our results synthetically characterize the hydrodynamics of the Mediterranean and Black Seas, complex and variable oceanic systems that function on multiple temporal and spatial scales [47]. Mesoscale features in the Mediterranean are an important source of variability [48], and we find that they are an important component to include in this bioregionalization. Indeed, the omission of mesoscale features and their temporal variability in such spatial analyses are misleading, as already suggested by [20], who promoted the use of dynamical biogeochemical subprovinces instead of their static equivalents. Classical features here are stable and are clearly representative of the biogeochemistry of the subprovince that they describe. Though highly variable, mesoscale features enable us to further discriminate additional regions. This is especially useful in the open ocean, which appears homogenous when considering only classical variables.

Overall, the biogeochemical subprovinces defined for the full array of variables (Figure 3c) can be organized into four broad categories that compare relatively well with previous studies. Subprovinces 1 and 2 represent the open ocean regions of the southern basin. These two subprovinces are highly correlated in terms of their PC1 and PC2 (Table S2), indicating that both their classical and mesoscale features vary similarly and appear to differ mostly in their bathymetry. Subprovince 3 is representative of regions of particularly high mesoscale activity. This is clearly the case for the Alboran Sea, the Algerian Basin, the Strait of Sicily and the Ierapetra, Rhodes and Mersa-Matrouh gyres, as confirmed in other studies (e.g., [8], [22], [37], [47], [49], [50]). Mesoscale activity is also particularly high for the phenological regions in the Alboran Sea and the Strait of Sicily found by [8], who defined seven regions based on satellite ocean color. They found no apparent bloom pattern in these regions, which coincide primarily with full subprovince 3 (characterized by highly variable chl; Table 2). Subprovince 4 represents the coastal regions with both narrow and wide continental shelves. Finally, subprovince 5 represents oceanic gyres at high latitude, including the Lion Gyre, though the South Adriatic Gyre [50] is not included in this subprovince, as might be expected. The biodiversity hot spots identified by [7] using exploited fish distributions also show good spatial agreement with our subprovinces. They highlight the importance of the western Mediterranean shelves, the Alboran Sea, the Adriatic Sea, and the Levantine Basin, which coincide with our coastal and high mesoscale activity subprovinces. These subprovinces are characterized by high and highly variable chl, an indicator of primary productivity [51], which may be associated with the high ecological productivity and biodiversity here [52]. The coastal regions and the large oceanic gyres revealed in this study are consistent with the subdivisions found by [31] using only dynamical criteria, e.g., advection and dispersion schemes due to surface currents. This suggests that the horizontal circulation potentially explains a significant part of the basin-scale distribution of oceanic tracers such as SST and chl. The mesoscale variability of the oceanic circulation also controls their smaller-scale patterns (through the formation of SST and chl frontal gradients) and is thus responsible for the lower stability of the boundaries in regions where mesoscale features are particularly ubiquitous.

The synthetic indices developed in this study via PCA show temporal variability at seasonal and interannual time scales. The seasonal signal of oceanographic conditions in the Mediterranean that is the dominant signal in all PCs is generally related to changes of heat and momentum fluxes, which also vary at seasonal time scales driven by synoptic weather patterns [53]. However, interannual variability, as shown to be strong for PC1 and 2, is often more complex and puzzling. Our attempt to explain the interannual signal that we found in PCs 1 and 2 showed no clear relationship to large-scale climate indices, which supposedly represent external atmospheric forcing over the basin [45], [46]. Though it is possible that our indices are not sufficiently long (101 months) to reveal any relationship to low-frequency signals, other potential drivers may help explain the interannual variability that we find. Among these are internal nonlinear ocean dynamics (e.g., unstable mesoscale eddy fields) that alter water properties and movements, the region and timing of deep-water formation, the extreme events and long-term variability of atmospheric forcing (e.g., winds, solar radiation, precipitation) that impact both the surface and sub-surface circulation, or interannual variations in the Gibraltar inflow [50].

The oceanographic variables defined here were included to capture as much of the ocean variability and dynamics as possible; however, our analysis indicated that some variables may be redundant. As noted, chl and chl frontal gradients are highly positively correlated (r = 0.92) and are almost always aligned with the same axis (Figure 5b–d arrow plots, Table S1, Table 3). For PC2, FSLE and EKE could also be redundant in that they are consistently aligned with the same axis for subprovinces 1–4 and are significantly negatively correlated (r = −0.53, p<0.001). This is consistent with the compact relationship (power-law) found between FSLE and EKE in the global ocean [54], although it appears less robust in the Mediterranean [55]. OW does not appear to be a useful variable to include in this bioregionalization, as it does not significantly contribute to the explanation of any axis (Table 3). This may be because OW is related to EKE and FSLE [55] and may be redundant in this analysis. In addition, it may exist at a lower spatial resolution than that detected by the 5% threshold level. At the 1% threshold level, it appears that OW may play a more important role in the bioregionalization (Figure S2b, c in File S1). Finally, frontal structures themselves indicate the boundaries between two different water masses [56] and between macro- or meso-provinces [33], making these variables potentially redundant to a partitioning analysis. However, this is not obvious *a priori*, as there is clearly a complex relationship between chl and chl fronts as indicated by their high correlation. We find that the contribution of SST and chl fronts is not strong or consistent in the Mediterranean or Black Seas.

In this study, we characterize the biogeographical conditions of the Mediterranean Sea in a simple and synthetic approach. We develop objective biogeochemical subprovinces that agree with spatial characterizations made in previous studies and are specifically adapted to be useful as geographic reference points in a management context. Our results highlight the importance of mesoscale features to help delineate further regions in the seemingly homogeneous open ocean. We suggest that these subprovinces could be relevant for defining pelagic habitats for marine protected areas that are dynamic, yet predictable enough to be important for foraging and breeding aggregations [57]. In addition, the synthetic indices developed here represent a baseline from which variability and future changes of important classical and mesoscale features can be assessed. An understanding of the fundamental ocean processes of these heavily-impacted bodies of water has important implications for climatic studies, anthropogenic impact mitigation, and marine resources management.

## Acknowledgments

We would like to thank NASA, NOAA, and AVISO for the freely available remotely-sensed data. We would also like to thank Francesco d'Ovidio for his expertise and FSLE code and Giorgio Caramanna for his help improving the manuscript.

## Supporting Information

File S1 **Supporting figures and tables.** Figure S1 in File S1: Boxplots of the bootstrapped (1000 times) between-clusters sum of squares divided by the total sum of squares (i.e., y-axis represents the proportion of the explained sum of squares) for *k* between 2 and 30 for k-means analyses performed on the (a) “classical”, (b) “mesoscale” and (c) “full” multivariate arrays. To identify the most appropriate *k* for each multivariate array, we define thresholds whereby the explained sum of squares for each additional *k* increases by less than 5% (red line) or less than 1% (blue line). Table S1 in File S1: The eigenvalues of each axis for the “full” multivariate array for biogeochemical subprovinces defined by the 5% threshold. To determine which principal components (PC) to retain, we used the common cutoff of eigenvalues ≥1. Figure S2 in File S1: Biogeochemical subprovinces of the Mediterranean Sea for the (a) “classical”, (b) “mesoscale”, and (c) “full” multivariate arrays using a 1% threshold on the explained sum of squares to define the optimal number of subprovinces (see text). Figure S3 in File S1: Spatial stability of the borders of biogeochemical subprovinces for the (a) classical, (b) mesoscale, and (c) full multivariate arrays. K-means analysis, using the *k* found in the time-averaged analyses (Table 1), are performed on the multivariate arrays at monthly time steps for the 101 months of the data set and using a 1% threshold on the explained sum of squares to define the optimal number of subprovinces (see text). Spatial stability is represented as the percentage of time that a boundary of the biogeochemical subprovinces is found at a particular pixel over the 101 months of the data set. Red colors indicate stable borders. Table S2 in File S1: Correlation coefficients between the retained principal components (PC) for each of the full biogeochemical subprovinces and the monthly anomalies of the large-scale climate indices: North Atlantic Oscillation (NAO), the East Atlantic pattern (EA), the East Atlantic-West Russia pattern (EAWR), and the Scandinavian pattern (SCAND). Only correlations above the 95% significance level are included. Table S3 in File S1: Correlation coefficients between the retained principal components (PC) for each of the full biogeochemical subprovinces. Significance levels are represented as p<0.001 ‘***’, p<0.05 ‘*’, not significant ‘ ’.
(DOC)
