# Supplementary material for: Defining Mediterranean and Black Sea Biogeochemical Subprovinces and Synthetic Ocean Indicators Using Mesoscale Oceanographic Features
Source: PLoS One. 2014 Oct 31;9(10):e111251. doi: 10.1371/journal.pone.0111251 (PMC4216069; doi:10.1371/journal.pone.0111251)
Supplement: File S1 — Supporting figures and tables. Figure S1 in File S1: Boxplots of the bootstrapped (1000 times) between-clusters sum of squares divided by the total sum of squares (i.e., y-axis represents the proportion of the explained sum of squares) for k between 2 and 30 for k-means analyses performed on the (a) “classical”, (b) “mesoscale” and (c) “full” multivariate arrays. To identify the most appropriate k for each multivariate array, we define thresholds whereby the explained sum of squares for each additional k increases by less than 5% (red line) or less than 1% (blue line). Table S1 in File S1: The eigenvalues of each axis for the “full” multivariate array for biogeochemical subprovinces defined by the 5% threshold. To determine which principal components (PC) to retain, we used the common cutoff of eigenvalues ≥1. Figure S2 in File S1: Biogeochemical subprovinces of the Mediterranean Sea for the (a) “classical”, (b) “mesoscale”, and (c) “full” multivariate arrays using a 1% threshold on the explained sum of squares to define the optimal number of subprovinces (see text). Figure S3 in File S1: Spatial stability of the borders of biogeochemical subprovinces for the (a) classical, (b) mesoscale, and (c) full multivariate arrays. K-means analysis, using the k found in the time-averaged analyses (Table 1), are performed on the multivariate arrays at monthly time steps for the 101 months of the data set and using a 1% threshold on the explained sum of squares to define the optimal number of subprovinces (see text). Spatial stability is represented as the percentage of time that a boundary of the biogeochemical subprovinces is found at a particular pixel over the 101 months of the data set. Red colors indicate stable borders. Table S2 in File S1: Correlation coefficients between the retained principal components (PC) for each of the full biogeochemical subprovinces and the monthly anomalies of the large-scale climate indices: North Atlantic Oscillation (NAO), the East Atlantic [file pone.0111251.s001.doc]

Supporting Information

Defining Mediterranean Sea biogeochemical subprovinces and synthetic ocean indicators using mesoscale oceanographic features

Anne-Elise Nieblas^1,*^, Kyla Drushka^2^, Gabriel Reygondeau^3^, Vincent Rossi^4^, Hervé Demarcq^5^, Laurent Dubroca^6^, Sylvain Bonhommeau^1^

^1^ Unité Mixte Recherche Ecosystèmes Marins Exploités 212, Institut Français de Recherche pour l’Exploitation de la Mer, Sète, France.

^2^ Applied Physics Laboratory, University of Washington, Seattle, Washington, United States.

^3^ Center for Macroecology, Evolution and Climate, National Institute for Aquatic Resources, Technical University of Denmark (DTU Aqua), Charlottenlund.

^4^ Instituto de FÍsica Interdisciplinary Sistemas Complejos, Institute for Cross-Disciplinary Physics and Complex Systems, (CSIC-UIB), Campus Universitat de les Illes Balears, Palma de Mallorca, Spain.

^5^ Unité Mixte de Recherche Ecosystèmes Marins Exploités 212, Institut de Recherche pour le Développement, Sète, France.

^6^ European Commission, Joint Research Center, Institute for Environment & Sustainability, Water Resources, Ispra, Italy.

* corresponding author: Anne-Elise Nieblas, [anne.elise.nieblas@gmail.com](mailto:anne.elise.nieblas@gmail.com)

Figure S1: Boxplots of the bootstrapped (1000 times) between-clusters sum of squares divided by the total sum of squares (i.e., y-axis represents the proportion of the explained sum of squares) for *k* between 2 and 30 for k-means analyses performed on the (a) “classical”, (b) “mesoscale” and (c) “full” multivariate arrays. To identify the most appropriate *k* for each multivariate array, we define thresholds whereby the explained sum of squares for each additional *k* increases by less than 5% (red line) or less than 1% (blue line).


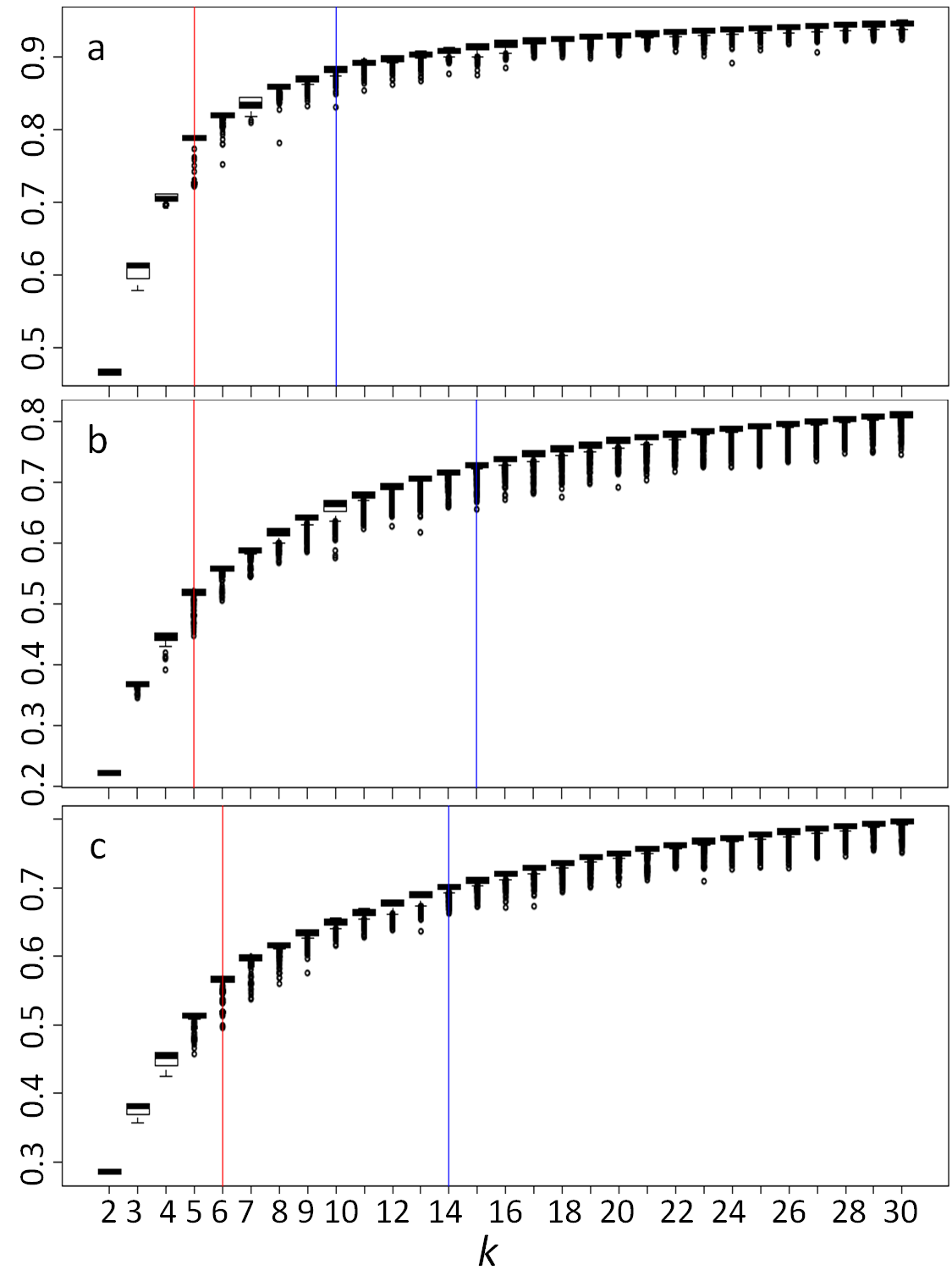


Table S1: The eigenvalues of each axis for the “full” multivariate array for biogeochemical subprovinces defined by the 5% threshold. To determine which principal components (PC) to retain, we used the common cutoff of eigenvalues ≥ 1.

| PC | subprovinces | | | | |
| --- | --- | --- | --- | --- | --- |
|  | 1 | 2 | 3 | 4 | 5 |
| 1 | 1.85 | 1.52 | 2.32 | 1.61 | 1.44 |
| 2 | 1.20 | 1.24 | 1.20 | 1.21 | 1.15 |
| 3 | 0.98 | 0.98 | 0.92 | 1.00 | 1.08 |
| 4 | 0.81 | 0.96 | 0.87 | 0.98 | 0.91 |
| 5 | 0.75 | 0.81 | 0.43 | 0.81 | 0.78 |
| 6 | 0.41 | 0.49 | 0.26 | 0.38 | 0.64 |


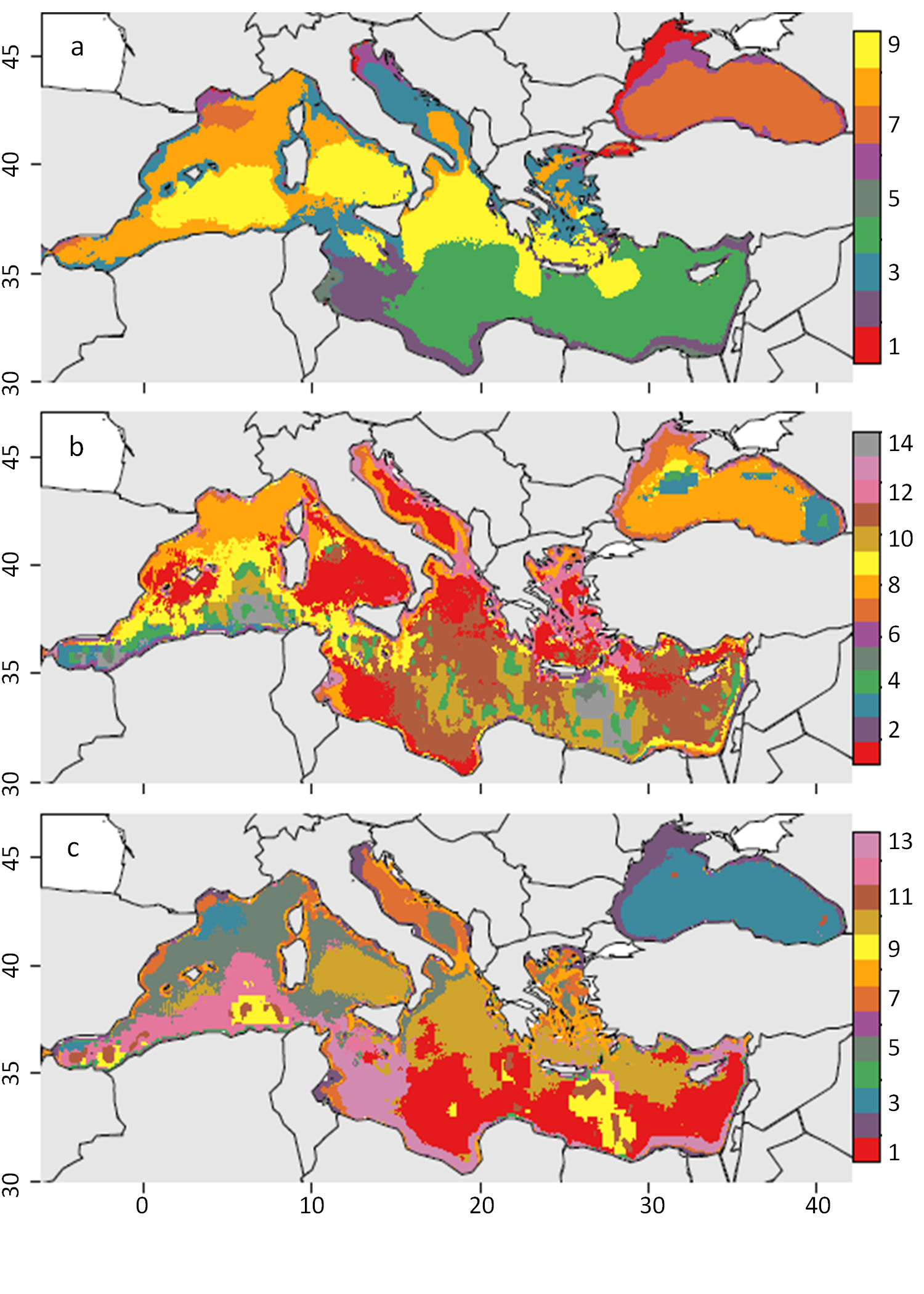


Figure S2: Biogeochemical subprovinces of the Mediterranean Sea for the (a) “classical”, (b) “mesoscale”, and (c) “full” multivariate arrays using a 1% threshold on the explained sum of squares to define the optimal number of subprovinces (see text).


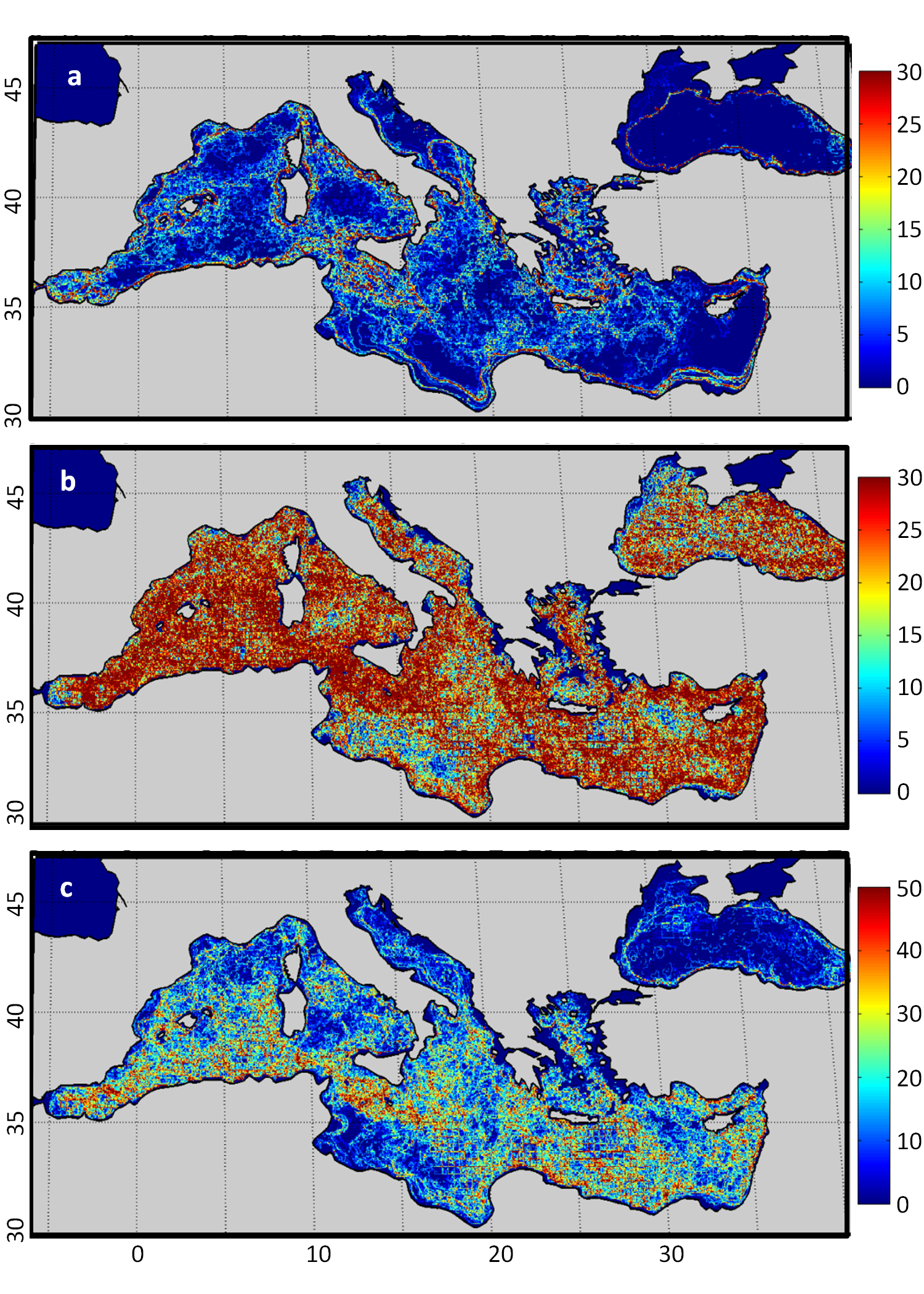


Figure S3: Spatial stability of the borders of biogeochemical subprovinces for the (a) classical, (b) mesoscale, and (c) full multivariate arrays. K-means analysis, using the *k* found in the time-averaged analyses (Table 1), are performed on the multivariate arrays at monthly time steps for the 101 months of the data set and using a 1% threshold on the explained sum of squares to define the optimal number of subprovinces (see text). Spatial stability is represented as the percentage of time that a boundary of the biogeochemical subprovinces is found at a particular pixel over the 101 months of the dataset. Red colors indicate stable borders.

Table S2: Correlation coefficients between the retained principal components (PC) for each of the full biogeochemical subprovinces and the monthly anomalies of the large-scale climate indices: North Atlantic Oscillation (NAO), the East Atlantic pattern (EA), the East Atlantic-West Russia pattern (EAWR), and the Scandinavian pattern (SCAND). Only correlations above the 95% significance level are included.

| PC | Climate indices | | | |
| --- | --- | --- | --- | --- |
|  | NAO | EA | EAWR | SCAND |
| Full subprovince 1 |  | | | |
| 1 |  |  |  |  |
| 2 |  |  |  |  |
| Full subprovince 2 |  | | | |
| 1 |  | 0.23 | 0.27 |  |
| 2 |  |  |  |  |
| Full subprovince 3 |  | | | |
| 1 |  | 0.21 |  |  |
| 2 |  |  |  |  |
| Full subprovince 4 |  | | | |
| 1 |  |  |  |  |
| 2 | -0.2 |  |  |  |
| 3 |  |  |  |  |
| Full subprovince 5 |  | | | |
| 1 | 0.23 |  | 0.22 |  |
| 2 |  |  |  |  |
| 3 |  |  |  |  |

Table S3: Correlation coefficients between the retained principal components (PC) for each of the full biogeochemical subprovinces. Significance levels are represented as p < 0.001 ‘***’, p < 0.05 ‘*’.

|  | PC1 | | | | |
| --- | --- | --- | --- | --- | --- |
| Subprovinces | 1 | 2 | 3 | 4 | 5 |
| 1 |  |  |  |  |  |
| 2 | 0.8 *** |  |  |  |  |
| 3 | 0.73 *** | 0.61*** |  |  |  |
| 4 |  |  |  |  |  |
| 5 |  |  |  |  |  |
|  | PC2 | | | | |
| Subprovinces | 1 | 2 | 3 | 4 | 5 |
| 1 |  |  |  |  |  |
| 2 | 0.58* |  |  |  |  |
| 3 |  |  |  |  |  |
| 4 |  |  |  |  |  |
| 5 |  |  |  |  |  |
|  | PC3 | |  |  |  |
| Subprovinces | 4 | 5 |  |  |  |
| 4 |  |  |  |  |  |
| 5 |  |  |  |  |  |
